# Integration of Multimodal Data from Disparate Sources for Identifying Disease Subtypes

**DOI:** 10.3390/biology11030360

**Published:** 2022-02-24

**Authors:** Kaiyue Zhou, Bhagya Shree Kottoori, Seeya Awadhut Munj, Zhewei Zhang, Sorin Draghici, Suzan Arslanturk

**Affiliations:** 1 Department of Computer Science, Wayne State University, Detroit, MI 48201, USA; kyzhou@wayne.edu (K.Z.); bhagyashree.k@wayne.edu (B.S.K.); seeya.munj@wayne.edu (S.A.M.); sorin@wayne.edu (S.D.); 2 Department of Electronic Engineering, Tsinghua University, Beijing 100084, China; 15111059@bjtu.edu.cn; 3 Department of Obstetrics and Gynecology, Wayne State University, Detroit, MI 48201, USA

**Keywords:** multimodal data fusion, imputation, deep learning, cancer progression

## Abstract

**Simple Summary:**

The diagnostic and treatment strategies of cancer remain generally suboptimal resulting in over-diagnosis or under-treatment. Though many attempts on optimizing treatment decisions by early prediction of disease progression have been undertaken, these efforts yielded only modest success so far due to the heterogeneity of cancer with multifactorial etiology. Here, we propose a deep-learning based data integration model capable of predicting disease progression by integrating collective information available through multiple studies with different cohorts and heterogeneous data types. The results have shown that the proposed data integration pipeline is able to identify disease progression with higher accuracy and robustness compared to using a single cohort, by offering a more complete picture of the specific disease on patients with brain, blood, and pancreatic cancers.

**Abstract:**

Studies over the past decade have generated a wealth of molecular data that can be leveraged to better understand cancer risk, progression, and outcomes. However, understanding the progression risk and differentiating long- and short-term survivors cannot be achieved by analyzing data from a single modality due to the heterogeneity of disease. Using a scientifically developed and tested deep-learning approach that leverages aggregate information collected from multiple repositories with multiple modalities (e.g., mRNA, DNA Methylation, miRNA) could lead to a more accurate and robust prediction of disease progression. Here, we propose an autoencoder based multimodal data fusion system, in which a fusion encoder flexibly integrates collective information available through multiple studies with partially coupled data. Our results on a fully controlled simulation-based study have shown that inferring the missing data through the proposed data fusion pipeline allows a predictor that is superior to other baseline predictors with missing modalities. Results have further shown that short- and long-term survivors of glioblastoma multiforme, acute myeloid leukemia, and pancreatic adenocarcinoma can be successfully differentiated with an AUC of 0.94, 0.75, and 0.96, respectively.

## 1. Introduction

Patients suffering from the same cancer disease may not only experience a high degree of symptomatic variability but also display significantly different responses to the same treatment. As a result, many cancers are over-diagnosed causing patients to receive unnecessary cancer treatments, while some patients do not receive the needed treatment. In addition, treatment responses vary significantly across different patients due to the complexity of cancer treatment. This can be greatly reduced by an early risk prediction model that can successfully differentiate between patients who are at higher-risk and need the most aggressive treatments from those who will never progress, recur, or develop resistance to treatments.

Studies have shown that different modalities (including gene expression, DNA Methylation, miRNA, variant data, lifestyle, clinical data), all play an important role towards predicting the development of cancer [1,2]. Van et al., López-García et al., Zhou et al., and Lu et al. have successfully classified tumors subtypes of Acute Lymphoblastic Leukaemia (ALL) using miRNA expression [3,4,5,6]. Lauber et al. identified short- and long-term survivors of Acute Myeloid Leukemia (AML) through *DNMT3A*, *FLT3* and/or *NPM1* mutations [7]. Similarly, Jonckheere et al. investigated that membrane bound mucin is responsible for short-term survival in patients with pancreatic adenocarcinoma [8]. Although such findings provide a better understanding and can help individualize treatment decisions, they may not provide a complete picture of the disease.

The human body consists of a mass of interconnecting pathways, working together in symphony. Output of one process, or a pathway, is further used by another process, or a pathway, for proper functioning of the body. Hence, deriving results based on just one modality, (e.g., gene expression) may not provide sufficient information. Plotnikova et al. and Jonas et al. discussed miRNA playing an important role in regulating gene expression [9,10]. Aure et al. studied the interaction of methylation and miRNA and their effect on predicting breast cancer [11]. This further suggests that the investigation of clinically relevant disease subtypes cannot be achieved by analyzing data from a single source. The proposed simulated study aims to achieve a novel data integration methodology that fuses information from disparate sources with different cohorts.

Data fusion refers to the integration of multiple modalities or data sources (i) to obtain a more unified picture and comprehensive view of the relations, (ii) to achieve more robust results, (iii) to improve the accuracy and integrity, and (iv) to illuminate the complex interactions among data features [12,13,14,15,16,17]. Nguyen et al. [18] have used a perturbation based data integration method to identify disease subtypes. Though powerful, these data integration methods often fail to generate stable results when high dimensional data with limited samples are present, mainly due to their randomized nature. End-to-end adversarial-attention network for multimodal clustering (EAMC) [19] allows the integration of multiple modalities through its discriminator module, which guides its encoders to learn a latent representation distribution by assigning one modality as an anchor to others. In the supervised learning setup, Wang et al. [20] performed a multimodal fusion by channel exchanging (CEN), which dynamically exchanges the features between different modalities to build inter-modal fusion for better segmentation or translation. Though powerful, these methods lack the ability to integrate data from heterogeneous sources with multiple modalities.

One straightforward approach to address the heterogeneous data sources’ integration is direct concatenation, which treats features extracted from different sources equally, by concatenating them into a feature vector. Treating different datasets equally by simply concatenating the features from disparate sources cannot achieve good performance due to different representation, distribution, scale, and density of data [21]. It also leads to challenges such as overfitting due to increased dimensionality of data after concatenation, as well as data redundancies and dependencies [22].

**Figure 1:**
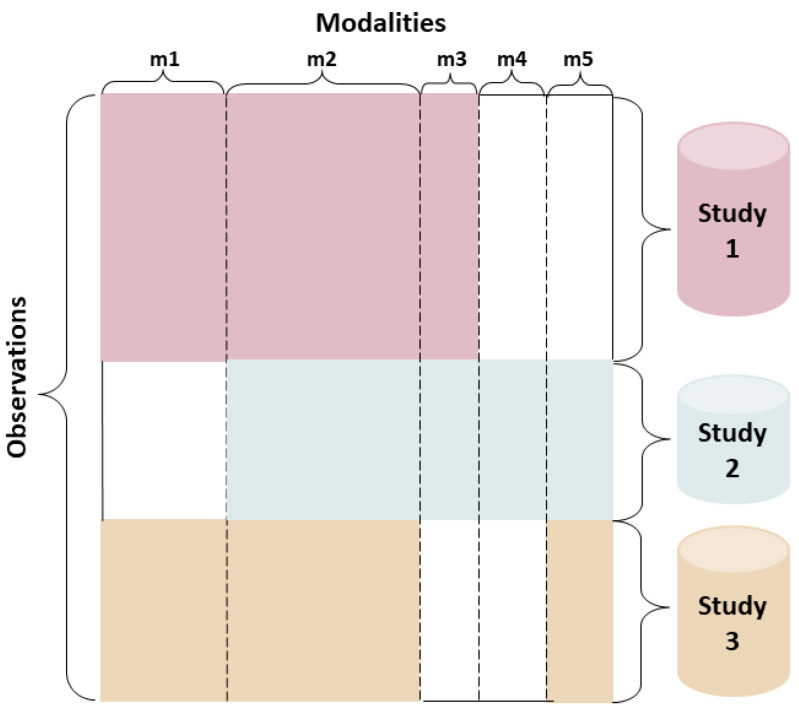
An illustration of data consisting of multiple sources with shared (i.e., m2) and unshared modalities (i.e., m1, m3, m4, and m5).

Multimodal data fusion [14] allows different datasets or different feature subsets of an object to be combined to describe the object comprehensively and accurately. The proposed research contributes to the young but growing field of multimodal data fusion with shared and unshared (modality-specific) data elements. Using heterogeneous datasets from disparate sources often lead to data blocks with partially shared features where observations (e.g., patients) from different sources differ in terms of the feature sets. Augmented multimodal setting allows an arbitrary collection of heterogeneous data sources to be partially coupled (i.e., one-to-one correspondence) through shared entities [23], which is illustrated in Figure 1. When multiple datasets (i.e., sources) with different modalities concerning an object exists, they cannot be simply merged into a single matrix for complementation of missing values due to each dataset having different distributions or feature dimensions. Traditional methods, such as coupled matrix and tensor factorization [24,25,26,27], and context-aware tensor decomposition [28], are used for joint matrix factorization analysis of partially coupled data from multiple platforms by transferring the similarity between object pairs learned from one dataset to the other for more accurate complementation of missing values.

Several alternative methods proposed for the complementation of missing values are summarized below. Yang et al. [29] proposed a semi-supervised learning approach to vote the predictions made by each modality individually. The incomplete modalities were filled with zeros, which may lead to biased predictions when high amounts of missing data are present. A computational approach based on deep neural networks to predict methylation states in single cells (DeepCpG) [30] was proposed to predict methylation states in single cells. Similarly, Yu et al. [31] imputed the missing DNA methylation values using a mixture regression model. Zhou et al. [32] proposed an autoencoder-like architecture that imputes the missing mRNA values by a nonlinear mapping from DNA methylation to gene expression data. The network was trained on a large scale pan-cancer dataset and then specifically fine-tuned for a targeted cancer through transfer learning. Bischke et al. [33] proposed a generative adversarial network to synthesize information from multiple modalities through a segmentation network. Ma et al. [34] proposed a reconstruction network, referred to as multimodal learning with severely missing modality (SMIL), that outputs a posterior distribution from which the missing modality is reconstructed through sampling using modality priors. With the priors learned from the existing modalities, such meta-learning framework predicts an embedding for missing modalities that can then be used for subsequent classification tasks. The cascaded residual autoencoder [35] was explored to impute the missing data by learning the complex relationship among certain modalities. This design required the network to compute the loss between each pair of cascaded autoencoder blocks, which may significantly increase the trainable parameters. Learning to recommend with missing modalities (LRMM) [36] is a controlled simulated study that randomly removes several features during training followed by the reconstruction of missing modalities through a generative autoencoder. This approach is similar to our proposed model, which is able to generate robust results, even with sparse or entirely missing modalities using image and textual data. However, the data used in this study contain spatial and/or temporal information that is mostly lacking in genomic data. Though powerful, classical data fusion approaches fail to integrate information from multiple data sources with disparate populations consisting of unshared modalities that are completely missing in one source while preserving the patient level information for further prediction tasks.

To address these issues, we propose a deep learning based data integration technique able to perform joint analysis on disparate heterogeneous datasets by discovering the salient knowledge of missing modalities. This is achieved by learning the latent association between existing and missing modalities followed by subsequent reconstruction of missing modalities. Our contributions are summarized as follows:

1. To the best of our knowledge, our approach is the first study that aims to discover the salient genetic knowledge of a completely missing modality through a mapping function learned by the neural network. This neural network model is able to reconstruct a lower dimensional representation of the missing information based on the correlation between shared and unshared modalities across data sources. Such mapping provides the ability to produce more accurate and consistent identification of aggressive and indolent patients for lethal cancers;
2. We have discovered patient subgroups and disease subtypes that have significantly different survival patterns through an unsupervised learning approach combined with manual adjustments which was then used for labeling the samples;
3. We quantitatively demonstrate that our work outperforms other baselines with partially available modalities.

In this paper, due to the small sample size, we adopt an autoencoder-like framework [37,38] for the compression and reconstruction of each modality. The lower dimensional latent representation will be used for the classification tasks. Similar to SMIL, we train our network to learn the approximated priors of the missing modality with respect to existing modalities. In our approach, a modality will be completely missing during the inference phase. Another substantial difference from previous studies is that our design only contains dense layers as our data do not have any spatial or temporal information.

## 2. Method

In this study, we aim to integrate the knowledge from disparate sources with shared and unshared modalities for a multimodal classification task. As seen in Figure 1, the data can consist of multiple sources (i.e., studies) with heterogeneous populations. In this paper, we conduct a simulated study by defining two heterogeneous populations, namely the reference data and the target data. Both the reference and the target data may contain shared and unshared modalities. The reference data are considered as the model’s training set for learning associations between its shared and unshared modalities, and the target data are considered as the testing set where the representation of an unshared modality that is completely missing (within the test set) can be inferred through the trained model. We formally define the reference and target data as $M^{r}=\left\{x_{i}|x_{i}\in R^{d_{i}},i=1,2,3,\ldots \right\}$ and $M^{t}=\left\{x_{i}|x_{i}\in R^{d_{i}},i=1,2,\ldots \right\}$, respectively. In our simulated study, all modalities in $M^{r}$ namely mRNA, DNA methylation, and miRNA are present, and one modality in $M^{t}$ namely DNA methylation is absent. We assume that the ground truth labels (e.g., aggressive vs. indolent tumor) for the reference and target data, $Y^{r}$ and $Y^{t}$, are known.

In case when all modalities are available in the target data, a simple autoencoder-like network [38,39] can be trained for multimodal classification. Particularly, Zhou et al. [38] showed that such an autoencoder-based classifier can handle data with high-dimensionality and limited sample size. However, in practice, certain modalities can be completely missing preventing the construction of a robust classifier. Therefore, our objective is to learn a mapping between the shared and unshared modalities using other sources (i.e., our training data/reference), and predict a lower dimensional representation of the missing modalities within the testing set. As such, we propose the architectures in Section 2.1.

### 2.1. Learning Associations between Shared and Unshared Modalities

For the aforementioned purpose, we have designed three independent network architectures for different scenarios. Different from commonly seen studies, all layers in our proposed architectures are dense, as we only consider vector-like data. Moreover, the elements in a single vector do not have any spatial or temporal information. The detail of the fundamental baseline architecture is provided in Table 1, which will be discussed in Section 2.1.1.

Instead of imputing severely missing values as seen in several other studies (up to, e.g., 90%), we propose a method that can learn the embedding of a completely missing modality specific to a single source (i.e., DNA methylation in our study) through the knowledge available in the other existing data source(s), which will be discussed in Section 2.1.2.

#### 2.1.1. Complete Fusion Autoencoder

**Figure 2:**
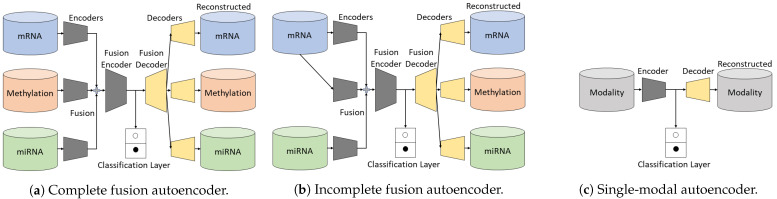
The overview of our proposed architectures. As the backbone, the complete fusion autoencoder (**a**) has three separate encoders for the modalities, i.e., encoder 1, encoder 2, and encoder 3. The fusion encoder then merges the learned representations from each thread for latter usage. Without losing generality, encoder 2 in (**b**) takes the first modality as input, to learn the correlation between mRNA and methylation. (**c**) simply takes every individual modality as input, respectively.

When all shared and unshared modalities are present, the complete fusion autoencoder (CFA) in Figure 2a is used to fuse the different inputs into a binding latent representation, which will then be decoded to reconstruct the corresponding modalities. The latent representation of the fused modalities is used for the classification task. For simplicity, we denote the encoders and decoders as single layers. The fused feature $Z=fuse(\left\{z_{i}|z_{i}\in R^{h_{i}},i=1,2,3\right\})$ highly compresses the prior knowledge of $\left\{x_{i}|x_{i}\in R^{d_{i}},i=1,2,3\right\}$ and is fed to another classification layer, where the fuse function is the concatenation or averaging operation, and $h_{i}$ denotes the hidden dimension of modality *i*. More specifically, the mapping *f* of $x_{i}\to z_{i}\to {\hat{x}}_{i}$ can be denoted as $z_{i}=act\left(W_{i}^{1}x_{i}+b^{1}\right)$ and ${\hat{x}}_{i}=act\left(W_{i}^{2}z_{i}+b^{2}\right)$, where $W_{i}^{1}\in R^{d_{i}\times h_{i}},b^{1}\in R^{h_{i}},W_{i}^{2}\in R^{h_{i}\times d_{i}},b^{2}\in R^{d_{i}}$, and act is the non-linear activation function (rectified linear unit (ReLU)). We apply 1D batch normalization on each block (consisting of dense and ReLU layers) in the encoders to alleviate internal covariate shift [40].

The optimizer of CFA utilizes the L2 loss as the reconstruction loss between each pair of $x_{i}$ and ${\hat{x}}_{i}$:

(1) $$L_{rec}=\sum_{i=1}^{M}\left|\right|x_{i}-{\hat{x}}_{i}{\left|\right|}_{2}^{2},$$

where *M* is the number of possible modalities (three in our study).

For each modality, let *p* and *q* be the prior and posterior distributions, $\exists \;x_{i}\;s.t.\;p\left(z_{i}\right)\approx p\left(x_{i}\right)$, where $p(x_{i})$ is hard to measure. Given the available modalities, $p(z_{i})$ can be approximated by $p_{\phi }\left(z_{i}\right)$ through Equation (1), where ϕ denotes the trainable parameters in the encoders and decoders.

#### 2.1.2. Incomplete Fusion Autoencoder

In the event that an unshared modality (e.g., $x_{2}$) is completely missing in the testing set, we train the network with the reference data to learn a mapping function $f^{\prime }$ of $x_{1}\to z_{2|1}\to {\hat{x}}_{2}$, where $z_{2|1}$ denotes the hidden feature representation of ${\hat{x}}_{2}$ given $x_{1}$. The reconstructed output ${\hat{x}}_{2}$ is optimized using the L2 loss according to $x_{2}$. As there are still reconstructions for the existing modalities ($x_{1}\to {\hat{x}}_{1}$ and $x_{3}\to {\hat{x}}_{3}$), we name this network as incomplete fusion autoencoder (IFA) for better illustration. The framework of IFA is shown in Figure 2b.

In CFA, all $p(x_{i})$ can be approximated by *f*. However in IFA, $∄\;x_{2}\;s.t.\;p\left(z_{2}\right)\approx p\left(x_{2}\right)$. Therefore, we use an encoder to learn the approximation $p(x_{2}|x_{1})$ through $f^{\prime }$, such that:

(2) $$\begin{array}{cc} p(x_{2}) & \approx p\left(x_{2}|x_{1}\right)\approx p\left(z_{2}|x_{1}\right)\approx p\left(z_{2}\right)\\ \; & \approx p_{\phi }\left(z_{2|1}|x_{2|1}\right)\approx p_{\phi }\left(z_{2|1}|x_{1}\right), \end{array}$$

where the true posterior $p(z_{2}|x_{1})$ is estimated by the distribution of $p_{\phi }\left(z_{2|1}|x_{1}\right)$ which is learned by the network, representing the prior distribution of the missing modality, i.e., $p({\hat{x}}_{2})$. We assume that Equation (2) is satisfied if $x_{2|1}$ highly correlates with $x_{1}$.

The procedure for calculating a mapping function $f^{\prime }=x_{1}\to z_{1|2}\to {\hat{x}}_{2}$ is described as $z_{2|1}=act\left(W_{2}^{1}x_{1}+b^{1}\right)$, ${\hat{x}}_{2}=act\left(W_{2}^{2}z_{2|1}+b^{2}\right)$, where $W_{2}^{1}\in R^{d_{1}\times h_{2}},b^{1}\in R^{h_{2}},W_{2}^{2}\in R^{h_{2}\times d_{2}}$. Since ${\hat{x}}_{2}$ is still obtained in this thread, the reconstruction loss is same as Equation (1). The other threads for modality 1 and 3 remain the same as in Section 2.1.1.

#### 2.1.3. Single-Modal Autoencoder

A single-modal autoencoder (SMA) is a standard autoencoder. We use such a network to perform baseline studies with respect to each single modality, as shown in Figure 2c. The number of units in each network may slightly differ for different modalities due to different data dimensions.

#### 2.1.4. Classification Layer

All three architectures described above have a classification layer for the prediction task, which consists of a dense layer and a sigmoid layer. We adopt the commonly used cross entropy loss for this classification task:

(3) $$L_{ce}=-\sum_{j=1}^{C}Y_{j}log\left(Y_{j}^{\prime }\right),$$

where $Y_{j}$ denotes the ground truth label, $Y_{j}^{\prime }$ denotes the predicted probability of the j’th class, and *C* denotes the number of classes. Particularly for IFA, let *g* be a function in this classification layer, such that $Y^{\prime }=g\left[f\left(x_{1}\right),f^{\prime }\left(x_{1}\right),f\left(x_{3}\right)\right]$, where $f^{\prime }$ maps $x_{1}$ to the posterior distribution of the missing modality $x_{2}$ based on the assumption made in Equation (2).

### 2.2. Joint Loss Optimization

As the weights in the autoencoder and classification layer are updated simultaneously, the objective is to minimize the joint loss:

(4) $$L_{joint}=\alpha L_{rec}+\beta L_{ce},$$

where α and β are the ratios for each loss. We empirically set them both to 1.

## 3. Experiments

In order to evaluate the effectiveness of the proposed IFA, we conduct several experiments using glioblastoma multiforme (GBM), acute myeloid leukemia (LAML), and pancreatic adenocarcinoma (PAAD) datasets by predicting patients’ disease progression status (short- vs. long-term survivors) and report the balanced accuracy and ROC AUCs.

### 3.1. Data Preparation

Here, we use the preprocessed GBM, LAML, and PAAD data (the three datasets or cancers hereafter) from The Cancer Genome Atlas (TCGA) as fully controlled simulated studies. There are a total of 273, 143, and 175 patients in the three datasets, respectively, each containing three separate modalities (mRNA, DNA Methylation, and miRNA). The data are normalized along the feature dimensions in all our analyses. In order to mimic the situation that one of the modalities is completely missing, for each of these cancers, we randomly select 36% of the samples as our testing set and remove their associated $m_{2}$ (i.e., DNA methylation) modality, keeping only $m_{1}$ (i.e., gene expression) and $m_{3}$ (i.e., miRNA). The remainder of samples along with their three modalities ($m_{1}$, $m_{2}$, and $m_{3}$) are reserved for training. Due to the limited sample sizes, we only simulate circumstances of two data sources in this study. The dimensions of $m_{1}$, $m_{2}$, and $m_{3}$ are shown in Table 2.

**Figure 3:**
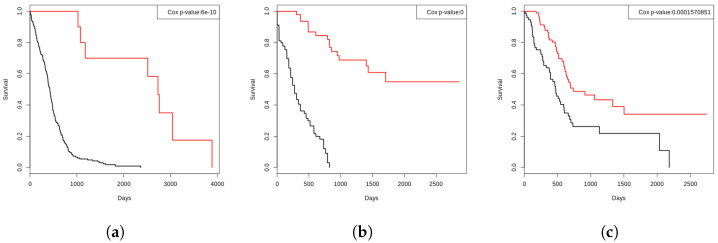
The Kaplan–Meier survival curves of the three cancers generated using our labeling strategy, indicating the reliability of our ground-truth labels. Here the black curves are representing the short-term survivors and the red curves are representing the longer-term survivors. (**a**) GBM. (**b**) LAML. (**c**) PAAD.

Our goal is to understand disease progression by classifying patients as short- and long-term survivors at the time of diagnosis and the approach discussed below is used to define labels for each sample in the train and validation sets for model building. In order to annotate the samples as short- and long-term survivors (or aggressive vs. indolent), we rely on an unsupervised learning algorithm, referred to as perturbation clustering for data integration and disease subtyping (PINS) [18], that is shown to be effective in subtype discovery using molecular data. PINS utilizes a consensus-like voting mechanism to select the best number of clusters among all modalities based on the k-means algorithm. The agreement between modalities is calculated to identify sub-populations through a hierarchical clustering approach. Next, we make some manual corrections on several observations that are classified incorrectly (outliers). For instance, if a GBM patient has lived only for 203 days after diagnosis, and is clustered as a long-term survivor by PINS, we manually correct that subject’s class label. After such minor corrections, we achieve two patient subgroups for each cancer annotated as short- and long-term survivors. The subtypes are validated using Kaplan–Meier analysis, and their statistical significance is assessed using Cox regression. The survival curves in Figure 3 show a clear and statistically significant separation between two groups of patients after the manual setup of labels generated through PINS, indicating the reliability of our ground truth labeling. After annotation, we end up having two groups, i.e., aggressive and indolent samples for different cancers as shown in Table 2.

### 3.2. Implementation Details

We implement our networks in PyTorch 1.4.0 with NVIDIA Titan RTX GPU. The Adam optimizer with a learning rate of 0.01 is used for training. All proposed models are trained for 100 epochs. The simple yet effective design of our network resulted in a run time of less than 2 min for a 5-fold cross validation model.

### 3.3. Correlation between Shared and Unshared Modalities

**Figure 4:**
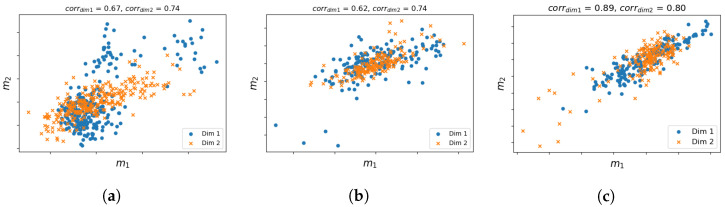
Visualization of the correlation between $m_{1}$ and $m_{2}$ with dimension reduction. Different markers represent the first and second dimensions. The two modalities are highly correlated as the points lie around the first diagonal. (**a**) GBM. (**b**) LAML. (**c**) PAAD.

In Section 2.1.2, we discussed that the prior approximation of a missing modality can improve the final prediction if there exists a high correlation between shared and unshared modalities. For this reason, we apply the partial least squares (PLS) algorithm [41] to first obtain the maximized cross-covariance matrix between shared and unshared modalities (i.e., $m_{1}$ and $m_{2}$), from which a Pearson correlation coefficient is calculated to indicate how well the two modalities are correlated. Figure 4 visualizes the PLS canonical correlation between the two modalities of the three cancers with reduced dimensionalities. The correlation scores are 0.67 and 0.74, 0.62 and 0.74, 0.89 and 0.80, respectively, for each dimension. The high correlations indicate that it is indeed possible to learn the knowledge of an unshared missing modality in the target dataset through a mapping function learnt from the associations between shared and unshared modalities within a reference dataset. Correlation between multiple modalities has been reported by several other studies [42,43].

### 3.4. Evaluation Metrics

We adopt the commonly used evaluation metrics in our experiments including balanced accuracy and ROC AUC.

### 3.5. Prediction Performances

We conduct a 5-fold cross validation to compare the predictive performances of the several proposed network architectures with other baselines. Regardless of the small sample sizes, all baselines produce desirable performances due to the effective feature compression fulfilled by Equation (1). The testing samples are selected according to different random seeds. First, we report the prediction performance of all actual modalities integrated ($m_{1}$, $m_{2}$, and $m_{3}$) using the complete data (i.e., the ground truth) through our CFA architecture. Next, the $m_{2}$ modality is removed, and the IFA architecture is used to learn a fused representation of the actual $m_{1}$ and $m_{3}$, combined with the predicted $m_{2}$. The fused representation is then used to predict the disease progression and the prediction performances are reported. Next, the prediction performance of the two modalities, i.e., $m_{1}$ and $m_{3}$ using the CFA architecture is reported, which is denoted as CFA-2M. Moreover, we directly report the performance of every single modality separately using our baseline model SMA. We do not compare our CFA (with all modalities) with previous deep learning based multimodal classification methods due to the lack of spatial information. CEN [20] also supports vector-like data by setting the parameters of height and width to 1. However, we do not compare our results with CEN as complete multimodal classification is not our primary focus.

**Figure 5:**
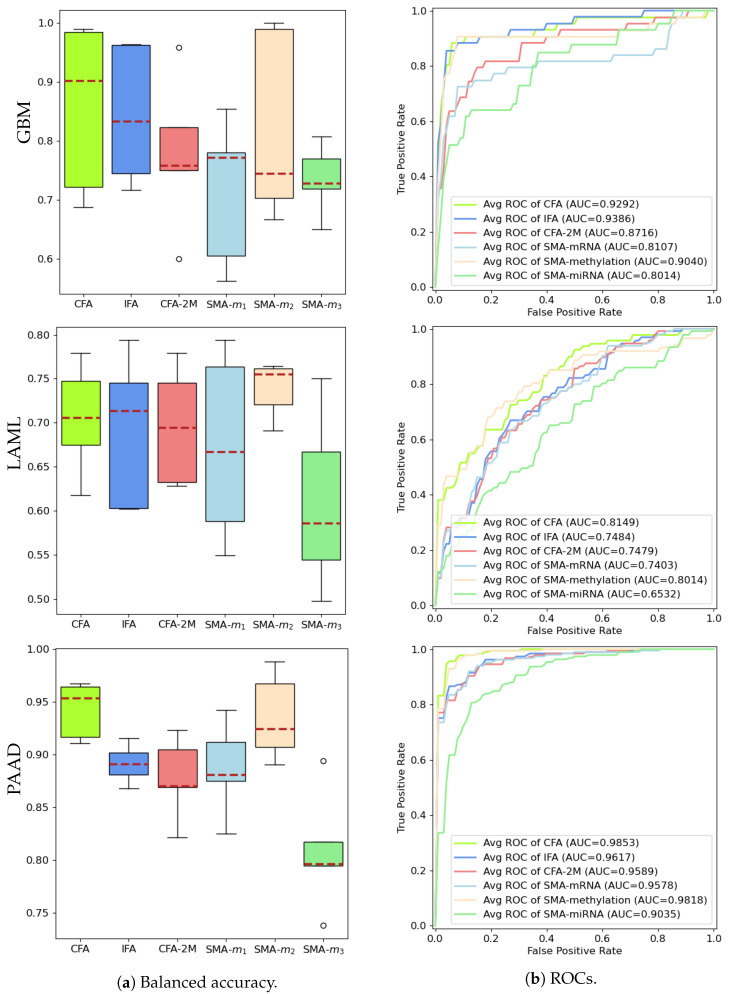
Comparison of balanced accuracy and ROC performances among the proposed IFA and all baseline predictors.

Figure 5 shows that inferring the highly predictive missing modality through the proposed data fusion model allows the construction of a predictor (IFA) that achieves comparable performances to CFA using all actual modalities, and is better than the baseline models that use only the two available modalities (CFA-2M) or a single modality (SMA-$m_{1}$, SMA-$m_{3}$). Since we have constructed a fully controlled simulated study, we are able to demonstrate the performance of SMA-$m_{2}$ (and, hence, CFA) in Figure 5a, which in practice would be absent. Results have further shown that certain modalities (i.e., DNA methylation, also referred to as SMA-$m_{2}$) carry more predictive information than others on specific cancers as can be seen in Figure 5a (for LAML and PAAD). The proposed study, therefore, is able to learn the latent representation of a modality with strong predictive capability that is completely missing in one source through another data source that carries shared entities. Specifically, the area under the ROC curve (AUC) reported in Figure 5b is calculated based on the averaged TPR values (across 5-fold runs) and linearly interpolated FPR values. Both balanced accuracy and AUC performances of our proposed models confirm the IFA’s ability to successfully fuse the knowledge learned from the missing modality. A t-test is applied to compare the performances of IFA with CFA-2M, SMA-$m_{1}$, SMA-$m_{3}$ across 100 runs on GBM and the results further confirmed that the distributions of balanced accuracies are significantly different with p-values of 0.04, <0.0001, and <0.0001, respectively. Similar significance levels are observed when the IFA models are compared with other baselines on LAML and PAAD except for LAML in which no significant difference between IFA and CFA-2M is noted.

### 3.6. Effective Compression

**Figure 6:**
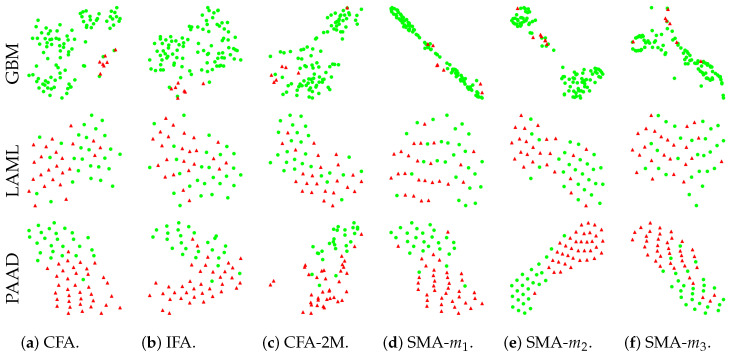
The t-SNE plots for compressed latent representations of proposed IFA and other baseline methods.

We show the t-SNE plots in Figure 6 for the compressed (and fused for further multimodal prediction) features in our baseline models. The same testing sets from one of the 5-fold cross-validation analyses are used for fair comparison across models. The plots show that the two patient subgroups in the latent space are well separated for both CFA (Figure 6a) and IFA (Figure 6b) models, while CFA-2M (Figure 6c) performs less optimal separation when compared with the other two models, indicating the proposed IFA’s ability to identify the short- and long-term survivors as accurately as CFA. For SMA using single modalities (Figure 6d–f), only the most predictive modality (i.e., DNA methylation) shows relatively well separated results as shown in Figure 6e. Figure 6 also indicates that our proposed models can accurately and robustly compress the multimodal and high-dimensional data with extremely limited sample size through conventional deep learning methods.

### 3.7. Functional Analysis

**Figure 7:**
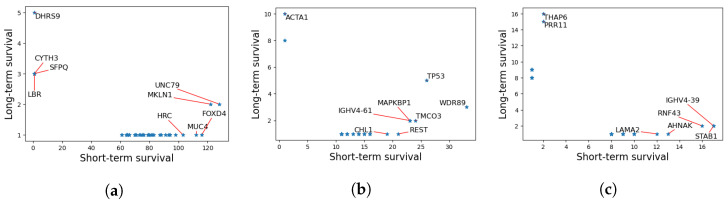
Number of patients in each group for each mutated gene in the three cancers. The *x*-axes represent the count for short-term survivors, and the *y*-axes represent the count for long-term survivors. Interesting genes appear in the lower right or upper left corners. (**a**) GBM. (**b**) LAML. (**c**) PAAD.

We have further identified mutations that are highly abundant in the short-term survival group, but not in the long-term survival groups for the three cancers. The results are reported in Figure 7. Here, each point represents a gene, and the coordinates are representing the number of patients having at least a variant in that gene for long- vs. short-term survivors. In principle, we are mostly interested in genes that are highly mutated in one group and not in the other which corresponds to the top-left and the bottom-right corners of the graph.

### 3.8. Ablation Study

An ablation study can be conducted without using a mapping function, i.e., by only using existing modalities. Such results are already demonstrated as CFA-2M in Figure 5.

## 4. Discussion

Data collection has been the principle bottleneck for advancement in the life sciences, particularly in genomics, engineering, and healthcare, it is not always possible to have access to different modalities collected from different populations. For instance, the disease progression prediction performance of pancreatic adenocarcinoma patients will significantly reduce when DNA methylation data are absent (as can be seen from Figure 5—performance of CFA-2M). The importance of DNA methylation on PAAD prediction is further supported by several studies, including Mishra et al. and Tan et al. [44,45]. The proposed integrative subtyping system, however, will circumvent the many challenges associated with missing modalities and the need to collect additional data by exploiting the current availability of vast genomic, epidemiologic, and clinical data.

Although deep learning models reach impressive prediction accuracies, their nested non-linear structure makes them highly non-transparent, i.e., it is not clear what information from the input data makes them actually arrive at their decisions. For clinicians, these models appear as “black boxes” and, hence, hamper their confidence in using them for clinical decision making, mainly because they are unable to compare to and integrate their expert opinion with the predictions. This, however, can be greatly reduced by explainable AI techniques that aims to understand how the model arrives at the decisions [46,47,48]. In this study, although we believe that the use of an autoencoder may lead to limitations in explainability, alternative models that exclude the encoding (i.e., predicting the actual values of a missing modality instead of a latent representation) would lead to curse of dimensionality issues given the high dimensional nature of genomic data with limited samples. Additionally, the use of autoencoders results in a lower dimensional latent representation of the data which prevents overfitting issues in subsequent prediction tasks.

In an effort to increase the usage in clinical practice, we examined the detailed mechanisms captured by our proposed classification model, for the three TCGA datasets, in terms of clinical variables, pathways, gene ontology (GO), and functional analysis. The more aggressive GBM subtype appears to affect mostly males with a 60% male dominance. The median age for the long- and short-term survivors are 35 and 60 years, respectively. GO analysis using iPathwayGuide (Advaita) suggests that the aggressive group has a stronger regulation of glial and astrocyte differentiation (p-values of 0.018 and 0.020) when compared to the less aggressive group. Chinnaiyan et al. [49] reported a similar phenomenon in aggressive glioma. Contrary to long-term survivors, the pathway analysis using iPathwayGuide (Advaita) has shown that the Phagosome pathway is significantly impacted (FDR corrected p-value: 0.037) on the short-term survivors group. Associations between Phagosome and glioblastoma has also been reported by Cammarata et al. [50].

Our PAAD and LAML classification results have shown that the classes are highly influenced by methylation profiles. For LAML, the median age for the long and short term survivors are 50 and 61 years, respectively. However, there is no dominance of age or gender in one group over the other in PAAD.

As seen in Figure 7, the short-term survival group is significantly rich in *MUC4*, *FOXD4*, and *HRC* mutations. *MUC4* is a transmembrane mucin that plays an important role in epithelial renewal and differentiation [51]. Several studies have identified associations between *MUC4* and GBM progression [52,53,54].

Similarly, we identified rich mutation counts in patients with aggressive LAML in genes including *TP53*, *TMCO3*, and *WDR89* and *REST*. According to Barbosa et al. although the tumor suppressor gene, TP53 has lower mutation frequencies in patients with LAML, such mutations are associated with high risk of relapse and resistance to treatment, which supports our findings [55].

Lastly, our findings has shown that several genes including *RNF43* and *STAB1* are reported to be associated with poor PAAD survival. *RNF43*, an E3 ubiquitin-protein ligase that acts as a negative regulator of the Wnt signaling pathway, is reported to be associated with various cancer types including PAAD [56]. On the contrary, *PRR11* is identified as a variant associated with long-term survival.

## 5. Conclusions

In this paper, we have presented a deep fusion model that is able to integrate knowledge of multiple studies with partially coupled data through shared entities. The proposed model is able to learn the knowledge of an entirely missing modality within one source through a mapping between the shared and unshared modalities within different sources. The results suggested that all modalities are functioning to the disease prediction, and are dependent on each other. Therefore, studying them together instead of separating them as independent sources of disease predictors, will provide more insights into the aggressiveness of the disease. We conducted several experiments using simulated data from the TCGA glioblastoma multiforme, acute myeloid leukemia, and pancreatic adenocarcinoma datasets. The proposed method enables a more robust and accurate prediction of the three cancers’ progression through integration, which is critical for making optimal treatment decisions.

Our models could be extended to other diseases if there exists correlation between the shared and unshared modalities. As a step towards overcoming the domain shift challenge, our approach has the potential to learn the complete knowledge of an unseen data source with missing modalities to improve the classification performance. Generally, our approach could be extended to more than two sources, as long as the additional testing set has the shared modality with the training set. As a future work, the integration of more than two separate data sources can be studied whenever more data are available.

The results of the proposed model can help optimize treatment by separating the patients with aggressive disease from those with less aggressive disease, as well as to increase the success of clinical trials by separating the respondents vs. non-respondents. The developed framework is expected to be a valuable precision medicine resource for the wider scientific community on other diseases.

## Figures and Tables

**Table 1:** The detailed number of units in the backbone architecture excluding the first and last layers.

Module	Neurons in Layer 1	Neurons in Layer 2
Encoder 1	1024	256
Encoder 2	1024	256
Encoder 3	64	-
Fusion Encoder	576	36
Fusion Decoder	512	-

**Table 2:** Dimensions of each modality ($m_{1}$, $m_{2}$,$m_{3}$) and the number of samples in each subtype for different cancers.

Cancer Type	Gene Expression ($m_{1}$)	DNA Methylation ($m_{2}$)	miRNA ($m_{3}$)	Short-Term Survival	Long-Term Survival
GBM	12,042	22,833	534	253	20
LAML	16,818	22,288	552	91	52
PAAD	14,105	20,006	257	75	100

## Data Availability

The results published here are in whole or part based upon data generated by The Cancer Genome Atlas managed by the NCI and NHGRI. Information about TCGA can be found at http://cancergenome.nih.gov, accessed on 1 September 2021. The source code and data are publicly available at https://github.com/ky-zhou/MMFDA, uploaded on 22 February 2022.
